# Associations between occupational stress and working conditions in a shoe and leather company

**DOI:** 10.1192/j.eurpsy.2023.2021

**Published:** 2023-07-19

**Authors:** N. Rmadi, I. Sellami, K. Jmal Hmmami, M. L. Masmoudi, M. Hajjaji

**Affiliations:** 1 Department Of Occupational Medicine, HEDI CHAKER hospital, University of Sfax, SFAX, Tunisia

## Abstract

**Introduction:**

Occupational stress (OS) is one of the major health hazards of the modern workplace. Poor working conditions are major occupational stressors and have a great impact on employees’ well-being.

**Objectives:**

This study aimed to assess the associations between OS and working conditions.

**Methods:**

We conducted an exhaustive cross-sectional study among workers in a shoe and leather company. We used the Job Demand Control model of Karasek to measure occupational stress. Workers were asked about their perception of working conditions such as the noise, heat generated by certain tools and machines, fabric smells and uncomfortable workspaces. Data were analysed using SPSS software.

**Results:**

The study involved 310 workers (58 men and 252 women) with an average age of 34.2 ± 10.3 years. Workers reported different concerns about working conditions. The noise was the major complaint reported by 73.7% of workers. Workspaces were uncomfortable according to 48.7% of workers. Job strain and isostrain situations were found in 56.5% and 44.5% respectively. Unpleasant smell from leather products was associated with job strain (p= 0.004, OR = 1.9, 95%; CI [1.2-3.1]) and isostrain (p= 0.043, OR = 1.6, 95%; CI [1.03-2.6]) situations. Heat generated by certain tools and machines was associated to isostrain situation (p= 0.009, OR = 2.7, 95%; CI [1.2-5.9]). Perceiving workspaces as uncomfortable was associated with isostrain situation (p= 0.004, OR = 1.9; 95%; CI [1.2-3.09]).

**Conclusions:**

Working conditions have an important impact on workers’ mental health. Thus, improving job conditions is a key way to improve workers’ health and well-being.

**Disclosure of Interest:**

None Declared

